# Serious adverse events in African–American cancer patients with sickle cell trait and inherited haemoglobinopathies in a SEER-Medicare claims cohort

**DOI:** 10.1038/s41416-019-0416-7

**Published:** 2019-03-20

**Authors:** Jessica R. Hoag, Biree Andemariam, Xiaoyan Wang, David I. Gregorio, Beth A. Jones, Jonathan Sporn, Andrew L. Salner, Helen Swede

**Affiliations:** 1 Department of Internal Medicine, Cancer Outcomes, Public Policy, and Effectiveness Research Center, Yale University School of Medicine, New Haven, CT USA; 2 New England Sickle Cell Institute, Division of Hematology/Oncology, Neag Comprehensive Cancer Center, UConn Health, Farmington, CT USA; 3 Center for Quantitative Medicine, UConn School of Medicine, Farmington, CT USA; 4 Department of Community Medicine and Health Care, UConn School of Medicine, Farmington, CT USA; 5 Department of Chronic Disease Epidemiology, Yale School of Public Health, New Haven, CT USA; 6 Department of Hematology-Oncology, St. Francis Hospital and Medical Center, Hartford, CT USA; 7 Hartford Health Care Cancer Institute, Hartford Hospital, Hartford, CT USA

**Keywords:** Prognostic markers, Cancer epidemiology

## Abstract

African–American (AA) cancer patients have long-experienced worse outcomes compared to non-Hispanic whites (NHW). No studies to date have evaluated the prognostic impact of sickle cell trait (SCT) and other inherited haemoglobinopathies, of which several are disproportionately high in the AA population. In a cohort analysis of treated patients diagnosed with breast or prostate cancer in the linked SEER-Medicare database, the relative risk (RR) for ≥1 serious adverse events (AEs), defined as hospitalisations or emergency department visits, was estimated for 371 AA patients with a haemoglobinopathy (AA+) compared to patients without haemoglobinopathies (17,303 AA−; 144,863 NHW−). AA+ patients had significantly increased risk for ≥1 AEs compared to AA− (RR = 1.19; 95% CI 1.11–1.27) and NHW− (RR = 1.23; 95% CI 1.15–1.31) patients. The magnitude of effect was similar by cancer type, and in analyses of AA+ with SCT only. Our findings suggest a novel hypothesis for disparities in cancer outcomes.

## Introduction

African–American (AA) cancer patients continue to exhibit worse survival compared to non-Hispanic whites (NHW) despite recent gains in all race/ethnic groups. Outcome disparities have been observed when controlling for socioeconomic status or under equivalent care in clinical trials, suggesting the presence of unmeasured clinical or intrinsic factors.^1,2^ An emerging area of investigation has focused on sources of adverse events (AEs) during anti-neoplastic treatment in AA cancer patients (e.g., renal toxicity, leukopenia). No prognostic studies to date have evaluated the import of inherited haemoglobinopathies or carrier states.^3^ Many of these conditions are disproportionately higher in the AA population, particularly so for sickle cell trait (SCT) with an estimated prevalence of 8% compared to <0.1% in NHWs. Contrary to the long-held consensus of the benign carrier genotype, emerging evidence shows higher risk for chronic renal dysfunction in the AA SCT population,^4^ as well as venous thromboembolism^5^ and ischaemic stroke^6^ compared to AA non-carriers. Evidence of underlying complications among other such inherited conditions (i.e., HbC, HbE, α-thalassemia and β-thalassemia trait) is more limited, but suggests associations with renal dysfunction^7^ and venous thromboembolism.^8^ Due to putative predisposing conditions, we hypothesise that physiological rigors of cancer therapy might trigger AEs among those with a haemoglobinopathy or carriers.

## Methods

### Study population

We analysed information from the SEER-Medicare database using a sample of fee-for-service enrollees aged 66 years or older being treated for the first instance of invasive breast (female) or prostate cancer diagnosed from 2007–2013 with follow-up through 2014. We restricted analyses to breast and prostrate cancers as they are the highest incident malignancies among AAs and NHWs. International Classification of Disease (ICD-9) codes were used to identify haemoglobinopathy patients and carriers (282.4–282.7). The analytic cohort consisted of *n* = 371 AA patients with a reported haemoglobinopathy/carrier state (AA+), and *n* = 17,303 (AA−) and *n* = 144, 863 (NHW−) without these conditions. The UConn institutional review board approved this study as exempt.

### Variables and statistical analysis

Serious AEs were defined as: hospitalisations based on the presence of a Medicare inpatient claim, and, emergency department (ED) visits using Current Procedural Terminology codes (99281–99285) within 12 months of treatment initiation. Prognostic tumour characteristics included stage, Gleason score (prostate), tumour size and number of positive lymph nodes. Comorbid conditions were derived from the Charlson Comorbidity Index (CCI). We used modified Poisson regression with robust error variance to estimate the relative risk (RR) for ≥1 serious AEs among AA+ compared to both AA− and NHW−. Based on unequal distribution of clinical and demographic characteristics across groups, we used three-way inverse probability of treatment weighting to estimate propensity scores to balance covariates across groups. We adjusted for census tract level socioeconomic data in the SEER file (i.e., median household income, percent with a high school education, rural/urban residence.) Analyses were performed with R Studio (Ver. 3.3.2) and SAS (Ver. 9.4).

## Results

**Fig. 1:**
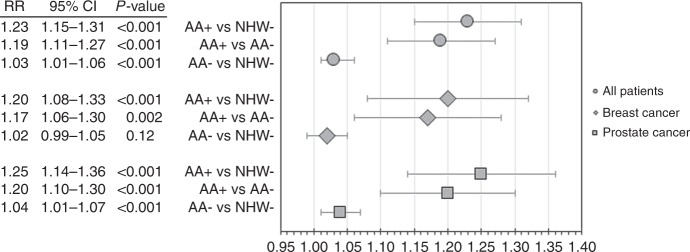
Propensity-weighted RRs and 95% CI for ≥1 Serious AEs in AA+ (n=371), AA- (n=17,303) and NHW- (n=144,863) Breast and Prostate Cancer Patients, SEER-Medicare Claims Data (2007-2013). Abbreviations: RR and 95% CI, relative risk and 95th % confidence interval; AE, adverse events (i.e., hospitalisations and/or emergency department visits); AA+, African-Americans with a haemoglobinopathy diagnosis; AA-, African-Americans without a haemoglobinopathy diagnosis; NHW-, Non-Hispanic whites without a haemoglobinopathy diagnosis

AA+ patients experienced a higher proportion of ≥1 serious AEs compared to AA− and NHW− patients (82.9%, 69.6%, 67.8%, respectively; *p* *<* 0.001), as well as ≥3 serious AEs (24.2%, 14.5%, 13.4%, respectively; *p* *<* 0.001; data not shown). In propensity score weighted Poisson regression (Fig. 1), AA+ patients had an increased risk for ≥1 serious AEs compared to both NHW− (RR 1.23, 95% CI 1.15–1.31, *p* < 0.001) and AA− (RR 1.19, 95% CI 1.11–1.27, *p* < 0.001). The magnitude and significance of these estimates were similar in breast and prostate cancer (Fig. 1), and when stratifying by haemoglobinopathy/carrier state (Table S1). AA− patients had slightly increased risk for serious AEs compared to NHW− patients in the full cohort and in prostate cancer (RR = 1.03, 95% CI 1.01–1.06, *p* < .001; RR 1.04, 95% CI 1.01–1.07, *p* *<* 0.001; respectively), but this effect did not reach it's significance in breast cancer (RR 1.02, 95% CI 0.99–1.05, *p* *=* 0.12). To address potential confounding given that AA+ patients had significantly more comorbid conditions compared to AA− and NHW− patients (Table S2), we performed a sensitivity analysis among patients with no reported comorbid conditions and found that the effect size for risk of serious AEs was consistent with our primary results (data not shown).

## Discussion

In this first analytic report, to our knowledge, we found that AA patients with a reported haemoglobinopathy/carrier state were more likely (17–25%) to experience at least one serious AE within 12 months following cancer-directed treatment than AA and NHW patients without these conditions. The magnitude of effect held for prostate cancer and female breast cancer alike, when stratifying by haemoglobinopathy/carrier state, and when examining a sub-sample of patients with no known comorbidities at diagnosis. Overall, our results are likely underestimates as we examined only major events (i.e., ED visits, hospitalisations). Lending further support for the impact of haemoglobinopathy/carrier states, the difference in outcome when comparing AA− with NHW− patients was quite small (2–4%).

Our results support observational evidence from large-scale studies showing an association of SCT with conditions possibly predisposing to treatment complications in cancer, particularly renal dysfunction, a disorder linked to poor prognosis in cancer patients.^9^ Cancer patients with SCT exhibiting established or sub-clinical kidney disease might be at increased risk for kidney-related complications when exposed to physiologic stressors of systemic chemotherapeutic agents and surgical anaesthesia. Another putative mechanism for an increased risk of AEs could be through alterations in erythrocyte shape and function resulting from hypoxia and other stressors, particularly in SCT. For example, reports show that erythrocytes from SCT carriers become more adhesive, a precursor to sickling crises, in response to *ex vivo* administration of the stress-related hormone epinephrine.^10^ Also, it is widely accepted that erythrocytes from SCT carriers are prone to sickling under conditions of extreme hypoxia, which can prompt a host of additional complications.

Our study has several important limitations. Specifically, the low prevalence of SCT in the cohort compared to population-based estimates underscores the inherent insufficiency of administrative claims for identifying genetic traits. Future studies with directly testing genotype are warranted. Additionally, the small sample size of AA+ cancer patients (*n* = 371) did not permit correlation of specific AEs with specific therapies nor stratification by major breast cancer subtypes which have varying prognoses and treatment protocols. Further, we were limited to census-tract level data of socioeconomic factors linked to disparities in cancer survival; information about relevant behavioural factors was unavailable; and, findings about Medicare enrollees might not be generalisable to younger cancer patients.

Our results could prompt discussion about adjustments to standard treatment plans, as well as adoption of closer monitoring of certain metabolic functions and haemoglobinopathy related adverse events (e.g., joint pain, deep-vein thrombosis). In the future, it could be informative to assess if haemoglobinopathy linked AEs partly account for higher rates of early discontinuation of cancer treatment observed in AA patients.

A current challenge in translating these findings to clinical practice is that most adult AA individuals along with their health care providers are unaware of their SCT status, unsurprising given that universal newborn screening was not fully implemented in the US until 2006. The value of genetic testing for haemoglobinopathies among AA cancer patients might merit discussion in the oncology community. Lastly, analyses of additional cancer types with established or putative outcome disparities may be justified to quantify the full prognostic import among the millions of cancer patients throughout the globe living in or descending from geographic regions noted for high prevalence of various haemoglobinopathies.

## Supplementary information

S.Table1

S.Table2

## Data Availability

SEER-Medicare database is publicly available
