# Supplementary material for: Serious adverse events in African–American cancer patients with sickle cell trait and inherited haemoglobinopathies in a SEER-Medicare claims cohort
Source: Br J Cancer. 2019 Mar 20;120(8):861–3. doi: 10.1038/s41416-019-0416-7 (PMC6474269; doi:10.1038/s41416-019-0416-7)
Supplement: Supplementary file 1 — S.Table1 [file 41416_2019_416_MOESM1_ESM.docx]

**Table S1 Relative Risk of ≥ 1 Serious AEs following Cancer-Directed Treatment by Hemoglobinopathy/Carrier Type**

|  | **SCT** | | | **SCD** | | | **Thalassemia** | | | **Other** | | |
| --- | --- | --- | --- | --- | --- | --- | --- | --- | --- | --- | --- | --- |
|  | **PS weighted RR** | **95% CI** | ***P*-value** | **PS weighted RR** | **95% CI** | ***P*-value** | **PS weighted RR** | **95% CI** | ***P*-value** | **PS weighted RR** | **95% CI** | ***P*-value** |
| ***All patients*** |  |  |  |  |  |  |  |  |  |  |  |  |
| NHW- | 1.00 | -- | -- | 1.00 | -- | -- | 1.00 | -- | -- | 1.00 | -- | -- |
| AA- | 1.10 | 1.09 - 1.12 | <.001 | 1.03 | 1.01 - 1.06 | <.001 | 1.03 | 1.01 - 1.06 | .002 | 1.03 | 1.01 - 1.06 | .002 |
| AA+ | 1.21 | 1.10 - 1.32 | <.001 | 1.31 | 1.20 - 1.43 | .002 | 1.30 | 1.19 - 1.41 | <.001 | 1.31 | 1.20 - 1.43 | <.001 |
| AA+ vs. AA- | 1.09 | 0.99 - 1.20 | .065 | 1.27 | 1.16 - 1.38 | <.001 | 1.25 | 1.15 - 1.38 | <.001 | 1.27 | 1.16 - 1.39 | <.001 |
| ***Breast Cancer*** |  |  |  |  |  |  |  |  |  |  |  |  |
| NHW- | 1.00 | -- | -- | 1.00 | -- | -- | 1.00 | -- | -- | 1.00 | -- | -- |
| AA- | 1.11 | 1.09 - 1.13 | 0.002 | 1.02 | 0.99 - 1.05 | .12 | 1.02 | 0.99 - 1.05 | .12 | 1.02 | 0.99 - 1.05 | .12 |
| AA+ | 1.20 | 1.07 - 1.35 | <.001 | 1.18 | 0.98 - 1.41 | .083 | 1.37 | 1.29 - 1.46 | <.001 | 1.31 | 1.17 - 1.46 | <.001 |
| AA+ vs. AA- | 1.08 | 0.96 - 1.22 | .19 | 1.15 | 0.96 - 1.38 | .14 | 1.34 | 1.26 - 1.43 | <.001 | 1.27 | 1.15 - 1.43 | <.001 |
| ***Prostate Cancer*** |  |  |  |  |  |  |  |  |  |  |  |  |
| NHW- | 1.00 | -- | -- | 1.00 | -- | -- | 1.00 | -- | -- | 1.00 | -- | -- |
| AA- | 1.10 | 1.08 - 1.12 | 0.02 | 1.04 | 1.01 - 1.07 | .004 | 1.04 | 1.01 - 1.07 | .004 | 1.04 | 1.01 - 1.07 | .004 |
| AA+ | 1.20 | 1.03 - 1.40 | <.001 | 1.41 | 1.28 - 1.54 | <.001 | 1.24 | 1.06 - 1.44 | .006 | 1.32 | 1.15 - 1.50 | <.001 |
| AA+ vs. AA- | 1.09 | 0.93 - 1.28 | 0.25 | 1.35 | 1.23 - 1.48 | <.001 | 1.19 | 1.02 - 1.38 | .028 | 1.26 | 1.11 - 1.44 | .001 |

***Abbreviations:*** AA+, African American with ≥ 1 hemoglobinopathy diagnosis; AA-, African American without a hemoglobinopathy diagnosis; NHW-, Non-Hispanic White without a hemoglobinopathy diagnosis; SCT, sickle cell trait, SCD, sickle cell disease; PS weighted RR, Propensity score weighted Relative Risk
