# Supplementary material for: Serious adverse events in African–American cancer patients with sickle cell trait and inherited haemoglobinopathies in a SEER-Medicare claims cohort
Source: Br J Cancer. 2019 Mar 20;120(8):861–3. doi: 10.1038/s41416-019-0416-7 (PMC6474269; doi:10.1038/s41416-019-0416-7)
Supplement: Supplementary file 2 — S.Table2 [file 41416_2019_416_MOESM2_ESM.docx]

**Table S2. Characteristics of Study Cohort by Race and Hemoglobinopathy/Carrier Status**

|  | **AA+** **n=371** | **AA-** **n=17,303** | **NHW-** **n=144,863** | ***P*-value^c^** |
| --- | --- | --- | --- | --- |
|  | **n (%)** | **n (%)** | **n (%)** |  |
| **Follow-up time**  Median months (IQR) | 52 (33-78) | 53 (32-77) | 57 (35-80) | <.001 |
| **Tumor site** |  |  |  |  |
| Breast (Female) | 187 (50.4) | 6,732 (38.9) | 68,714 (47.4) | <.001 |
| Prostate | 184 (49.6) | 10,571 (61.1) | 76,149 (52.6) |  |
| **Age** |  |  |  |  |
| 66-74 | 233 (62.8) | 10,554 (61.0) | 79,167 (54.7) | <.001 |
| 75-84 | 106 (28.6) | 5,335 (30.8) | 50,469 (34.8) |  |
| ≥85 | 32 (8.6) | 1,414 (8.2) | 15,227 (10.5) |  |
| **CCI Weighted Score** |  |  |  |  |
| 0 | 85 (22.9) | 6,467 (37.4) | 71,354 (49.3) | <.001 |
| 1-2 | 150 (40.4) | 6,861 (39.7) | 55,175 (38.1) |  |
| ≥3 | 136 (36.7) | 3,975 (23.0) | 18,334 (12.7) |  |
| **AJCC TNM Stage** |  |  |  |  |
| 0 | 34 (9.2) | 1,157 (6.7) | 10,620 (7.3) | <.001 |
| I | 67 (18.1) | 2,125 (12.3) | 29,281 (20.2) |  |
| II | 190 (51.2) | 10,242 (59.2) | 76,679 (52.9) |  |
| III | 21 (5.7) | 1,073 (6.2) | 9,641 (6.7) |  |
| IV | 37 (10.0) | 1,507 (8.7) | 9,359 (6.5) |  |
| Unknown | 22 (5.9) | 1,199 (6.9) | 9,283 (6.4) |  |
| **# Positive Lymph Nodes** |  |  |  |  |
| 0 | 108 (29.1) | 3,834 (22.2) | 45,804 (31.6) | <.001 |
| 1-3 | 19 (5.1) | 991 (5.7) | 8,713 (6.0) |  |
| >4 | 13 (3.5) | 475 (2.8) | 3,696 (2.6) |  |
| No nodes examined | 215 (58.0) | 11,445 (66.1) | 82,767 (57.1) |  |
| Unknown | 16 (4.3) | 558 (3.2) | 3,883 (2.7) |  |
| **Tumor Size (cm)^a^** |  |  |  |  |
| 0-2 | 91 (48.7) | 3,060 (45.5) | 38,564 (56.1) | <.001 |
| 2.1-3 | 64 (34.2) | 2,226 (33.1) | 19,984 (29.1) |  |
| ≥4 | 17 (9.1) | 639 (9.5) | 3,854 (5.6) |  |
| Unknown | 15 (8.0) | 807 (12.0) | 6,312 (9.2) |  |
| **Tumor Grade^a^** |  |  |  |  |
| Well differentiated (I) | 30 (16.0) | 1,072 (15.9) | 15,698 (22.9) | <.001 |
| Moderately differentiated (II) | 62 (33.2) | 2,522 (37.5) | 28,151 (41.0) |  |
| Poorly differentiated (III) | 65 (34.8) | 2,133 (31.7) | 16,973 (24.7) |  |
| Undifferentiated (IV) | N/A^d^ | 72 (1.1) | 861 (1.3) |  |
| Unknown | N/A | 933 (13.9) | 7,031 (10.2) |  |
| **Gleason Score^b^** |  |  |  |  |
| Well differentiated (2-4) | N/A | 110 (1.0) | 734 (0.96) | <.001 |
| Moderately differentiated (5-7) | 103 (56.0) | 5,770 (54.6) | 42,871 (56.3) |  |
| Poorly-differentiated (8-10) | 63 (34.2) | 3,959 (37.5) | 27,968 (36.7) |  |
| Unknown | N/A | 732 (6.9) | 4,576 (6.0) |  |

***Abbreviations***: AA+, African American with one or more hemoglobinopathies; AA-, African American with no hemoglobinopathy; NHW-, Non-Hispanic White with no hemoglobinopathy; IQR, Inter-Quartile Range; CCI, Charlson Comorbidity Index; AJCC, American Joint Commission on Cancer.

1. Breast Cancer
2. Prostate Cancer
3. Three-group comparison using Chi-square test.
4. Not available, values suppressed due to cell size n<11 as per SEER-Medicare data use agreement.
